# Possible role of glial cells in the relationship between thyroid dysfunction and mental disorders

**DOI:** 10.3389/fncel.2015.00194

**Published:** 2015-06-03

**Authors:** Mami Noda

**Affiliations:** Laboratory of Pathophysiology, Graduate School of Pharmaceutical Sciences, Kyushu University, 3-1-1 Maidashi, Higashi-kuFukuoka, Japan

**Keywords:** thyroid hormones, triiodothyronine, microglia, migration, phagocytosis

## Abstract

It is widely accepted that there is a close relationship between the endocrine system and the central nervous system (CNS). Among hormones closely related to the nervous system, thyroid hormones (THs) are critical for the development and function of the CNS; not only for neuronal cells but also for glial development and differentiation. Any impairment of TH supply to the developing CNS causes severe and irreversible changes in the overall architecture and function of the human brain, leading to various neurological dysfunctions. In the adult brain, impairment of THs, such as hypothyroidism and hyperthyroidism, can cause psychiatric disorders such as schizophrenia, bipolar disorder, anxiety and depression. Although impact of hypothyroidism on synaptic transmission and plasticity is known, its effect on glial cells and related cellular mechanisms remain enigmatic. This mini-review article summarizes how THs are transported into the brain, metabolized in astrocytes and affect microglia and oligodendrocytes, demonstrating an example of glioendocrine system. Neuroglial effects may help to understand physiological and/or pathophysiological functions of THs in the CNS and how hypo- and hyper-thyroidism may cause mental disorders.

## Introduction

Thyroid hormones (THs; Rothsschild et al., 2006) are critical for the development and function of the central nervous system (CNS; Zoeller and Rovet, 2004; Stenzel and Huttner, 2013). THs regulate development and differentiation of neurons and neuroglia (Gomes et al., 1999; Billon et al., 2001; Lima et al., 2001; Jones et al., 2003; Baxi et al., 2014; Dezonne et al., 2015). There are 2 major types of THs in the CNS represented by L-tri-iodothyronine (T3) and L-thyroxine (T4). The T4 is the major TH secreted by the follicular cells of thyroid gland; whereas T3, the most powerful TH, is mainly produced locally within the brain tissue by 5’-deiodination of T4. The T3 is an active form of the thyroid hormone (TH) essential for the development and function of the CNS.

Hyperthyroidism and hypothyroidism result from overactivation or suppression of thyroid grand leading to either excessive or insufficient production of THs. The prevalence of subclinical hyperthyroidism ranges from 1–15%, and of subclinical hypothyroidism from 3–16% in individuals aged 60 years and older was reported, which also suggested that there are differences in age, gender, and dietary iodine intake in the populations studied (Biondi and Cooper, 2008).

Any impairment of THs supply to the developing CNS causes severe and irreversible changes to the overall architecture and function of human brain, leading to various neurological dysfunctions (Di Liegro, 2008; Henrichs et al., 2010; Duntas and Maillis, 2013). Although in many respects the hypothyroid brain appears morphologically normal, clinical observations reported that hypothyroidism may be associated with both neurological and behavioral abnormalities as well as with functional impairments including mental retardation, ataxia and spasticity (Thompson and Potter, 2000). Psychiatric symptoms of hypothyroidism can include psychosis, mood instability, mania, hypersomnia, apathy, anergia, impaired memory mimicking dementia (Osterweil et al., 1992; Goh et al., 2014), psychomotor slowing, and attentional problems (Awad, 2000). The incidence of hypothyroidism increases with age, and adult-onset hypothyroidism is one of the most common causes of cognitive impairment (Mallett et al., 1995; Dugbartey, 1998).

On the other hand, hyperthyroidism may induce emotional lability, impatience and irritability, distractible overactivity, exaggerated sensitivity to noise, problems with sleep and the appetite (Awad, 2000) or depression and anxiety (Demet et al., 2002). Even at subclinical level, hyperthyroidism in the elderly is suggested to remarkably increase the risk of cognitive decline, dementia and Alzheimer’s disease (AD; Kalmijn et al., 2000; van Osch et al., 2004; Wijsman et al., 2013).

Multiple studies have reported that both hypo- and hyperthyroidism may potentially increase the risk of cognitive impairment and neurodegeneration. It has been also reported that both hyper- and hypothyroidism can affect the immune system (Klecha et al., 2008; De Vito et al., 2012). Hyperthyroidism decreases the proinflammatory activities of monocytes and macrophages. On the other hand, during hypothyroidism enhancement of phagocytosis and increased levels of ROS may occur so that the expression of proinflammatory molecules such as macrophage inflammatory protein-1α and interleukin-1β increases (De Vito et al., 2011). In contrast, hypothyroidism is reported to produce opposite effects on the immune function, such as decrease in immune response, antibody production, cell migration, and lymphocyte proliferation markers (Klecha et al., 2000, 2006), antioxidant enzymes and their activity (De Vito et al., 2011). The role of microglia, an immune cell population in the CNS, in this relationship between thyroid dysfunctions and neuropsychological disorders remains to be elucidated. In addition, knowledge of how other glial cells are involved in neuropsychological disorders, especially in the TH-sensitive regions of the brain (Fonseca et al., 2013), needs to be considered.

## Transportation of THs to The Brain and Metabolism in Astrocytes

**Figure 1:**
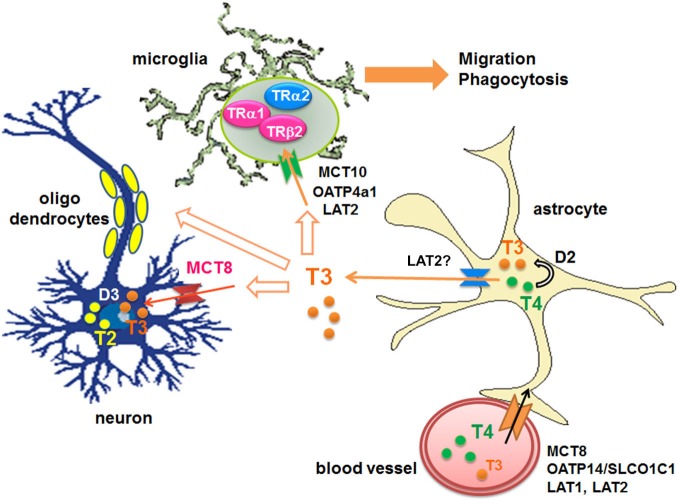
**Transport of THs to the brains and their metabolism**. D: iodothyronine deiodinases, LAT: L-type amino acid transporter, MCT: monocarboxylate transporter, OATP: organic anion-transporting polypeptide, T2: diiodothyronines, T3: triiodothyronine, T4: thyroxine, TR: thyroid hormone receptor.

Circulating T4 is transported across the blood-brain barrier via specific transporters such as organic anion-transporting polypeptides (OATPs) containing OATP14/SLCO1C1 (OATP1c1) (Sugiyama et al., 2003; Tohyama et al., 2004) and OATP1a2 (Gao et al., 2000; Lee et al., 2005; Hagenbuch, 2007), L-type amino acid transporters (LAT1 and LAT2), mainly LAT1 (Taylor and Ritchie, 2007), and monocarboxylate transporters 8 (MCT8) (SLC16A2) (for both T3 and T4) (Roberts et al., 2008). T4 also enters into astroytes through OATP1c1 (Dezonne et al., 2015), where it is de-iodinated by type 2-deiodinase (D2) to produce T3 (Guadaño-Ferraz et al., 1997; Fliers et al., 2006; Di Liegro, 2008). Subsequently T3 is released by LAT (Francon et al., 1989; Blondeau et al., 1993), presumably LAT2, and taken by other cells via distinct transporters; For example neurons express MCT8, while microglia express OATP4a1, LAT2 and MCT10 (Braun et al., 2011; Figure 1).

## Thyroid Hormone Receptors

The majority of TH effects are mediated through TH receptors (TRs), which belong to the members of the nuclear receptor superfamily and which function as T3-inducible transcription factors that are expressed in a tissue-specific and developmentally regulated manner (Cheng et al., 2010). In mammals, there are several TR isoforms: TRα1, TRα2, TRβ1, TRβ2a and TRβ3 (Koenig et al., 1989; Macchia et al., 2001). Among TRs, TRα1 is predominantly and widely expressed in the developing brain. The genomic actions of THs are exerted by the binding of T3 to nuclear TRs, which can either repress or activate gene expression.

Expression of TRα1 and TRβ1 have been identified in primary cultured rat microglia (Lima et al., 2001). Mutation of TRα1 in humans is associated with abnormal levels of TH but normal levels of thyrotropin as well as with growth retardation, and mildly delayed motor and cognitive development (van Mullem et al., 2012). A child with classic features of hypothyroidism with a *de novo* heterozygous nonsense mutation in a gene encoding TRα was also identified (Bochukova et al., 2012).

In addition to genomic effects of TRs, nongenomic signaling of THs through a plasma membrane–localized receptor has been recently described (Kalyanaraman et al., 2014; Mori et al., 2015). Potential mechanism for integrating regulation of development and metabolism by thyroid hormone and receptor tyrosine kinases through association of TRβ with PI3K was also suggested (Martin et al., 2014). These nongenomic effects of T3 may be important for glial function, which will be discussed later. In addition, it was suggested that the heterogeneity of TR expression throughout brain regions and between different cell types might lead to diverse effects on neuronal morphogenesis. The complexity may also result from not only the direct action of the hormone on neurons but also from indirect actions triggered by astrocytes (Dezonne et al., 2015) or other glial cell type.

## Effects of T3 on Microglia

Microglia, the resident macrophages of the CNS are generally considered the primary immune cells of the brain (Kim and de Vellis, 2005). In healthy CNS, ramified microglia are widely distributed to detect any environmental changes by their motile processes (Streit et al., 1988; Kettenmann et al., 2011). Pathological insults of multiple etiologies trigger microglial activation, represented by multi-stage and complex remodeling involving rapid migration towards the lesion site and phagocytosis of damaged cells (Becher et al., 2000; Hanisch and Kettenmann, 2007; Tanaka et al., 2009). It is generally recognized that the microglial phenotype may change depending on the microenvironment, which can be modified by various factors associated with specific types and stages of pathology (Perry et al., 1993; Scheffel et al., 2012; Solito and Sastre, 2012).

Microglial activation contribute to various pathologies (including, for example, Alzheimers disease) (El Khoury and Luster, 2008; Solito and Sastre, 2012). Recently, activated microglia have been indicated to cause also psychiatric disorders. Positron emission tomography imaging and postmortem studies have revealed microglial activation and abnormalities in schizophrenia, depression and autism (Kato et al., 2013; Mizoguchi et al., 2014; Monji et al., 2014).

**Figure 2:**
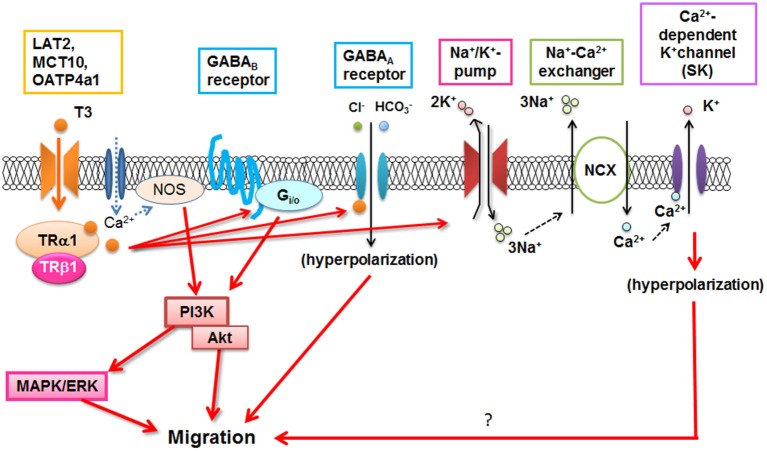
**T3-induced signal transduction in microglia**. NOS: Nitric oxide synthase, NCX: Na^+^/Ca^2+^ exchanger. (Modified from Mori et al., 2015).

T3 is important for microglial development (Lima et al., 2001), and could directly or indirectly stimulate morphological maturation of amoeboid microglial cells and limit their degeneration (Mallat et al., 2002). Recently, it has been demonstrated that T3 stimulates microglial migration and phagocytosis *in vitro and in vivo* (Mori et al., 2015). Microglial migration is mediated through T3 uptake by TH transporters and binding to the TRs. Then TH signaling in microglia involved several signaling pathways including G_i/o_-protein, PI3K, and MAPK/ERK, as reported in ATP-induced microglial migration (Honda et al., 2001). T3-induced nitric oxide signaling (Kalyanaraman et al., 2014) is also present in microglia (Mori et al., 2015). In addition, Na^+^/K^+^-ATPase, Na^+^/Ca^2+^ exchanger operating in the reverse mode, and GABA receptors contribute to T3-induced microglial migration (Figure 2; Mori et al., 2015), although the precise mechanism is still unknown. Since dysfunction of T3 in the aged brain significantly affected microglial morphology (Mori, 2014), microglial dysfunction may be closely related to psychological impairment in hypo- or hyper-thyroidism in elderly patients which will be investigated in the future.

## Astrocytes and Oligodendrocytes Differentiation by T3

In developing CNS T3 exerts numerous effects regulating axonal myelination and dendritic growth (Walravens and Chase, 1969; Legrand, 1982; Porterfield and Hendrich, 1993; Bernal and Nunez, 1995; Vose et al., 2013) and astrocyte and oligodendrocyte differentiation (Martinez-Galan et al., 1997, 2004; Jones et al., 2003; Schoonover et al., 2004; Manzano et al., 2007; Dezonne et al., 2009; Baxi et al., 2014). Effects of TH on astrocytes have been recently reviewed (Dezonne et al., 2015). Also, differentiation of human cultured CD34+ stem cells into oligodendrocyte precursors under THs action was also reported (Venkatesh et al., 2014). Expression alterations of genes using hypothyroidism model rats showed that immature astrocytes immunoreactive for vimentin and glial fibrillary acidic protein (GFAP) were increased, while oligodendrocyte lineage transcription factor 2 were decreased in the corpus callosum (Shiraki et al., 2014). Effects and molecular mechanisms of T3 action on astrocytes and oligodendrocytes in matured or aged brain remain to be investigated.

## Dysfunction of Glial Cells and Psychiatric Symptoms

As mentioned above, THs are not only important for neuronal development (Rami et al., 1986; Gould and Butcher, 1989) but it also support development of microglia (Lima et al., 2001), astrocytes (Gould et al., 1990; Manzano et al., 2007) including radial glial cells (Martinez-Galan et al., 2004), and oligodendrocytes (Walravens and Chase, 1969; Jones et al., 2003). Hypothyroid animals and TR mutant mice exhibit severe deficits in glial development (Morte et al., 2004). Therefore, indirect action of THs that occurs through astrocytes at different stages of brain development may contribute to neuronal progenitor proliferation, neuronal migration and differentiation, axonal growth and synapse function (Lima et al., 1998; Gomes et al., 1999; Martinez and Gomes, 2002; Martinez et al., 2011; Dezonne et al., 2013). Therapeutic use of THs in psychiatric disorders, mainly in depression, came to the light thus contributing to better understanding the action of THs in the brain (Weissel, 1999). Perhaps the major role of thyroxine therapy on depression might be due to hypothalamus-pituitary-thyroid axis activity and serotonin function in depressive episodes (Gomes et al., 2001). Neuroglial cells, as well as neurons contribute to psychiatric symptoms. For example, activated microglia and astrocytes in immunologically induced fatigue (Ifuku et al., 2014), microglial oxidative reactions in schizophrenia (Kato et al., 2011; Monji et al., 2013), and alteration of astrocytes or oligodendrocyte function in bipolar disorder (Dong and Zhen, 2015) have been reported. On the other hand, decreased glial density in association with glial hypotrophy in bipolar disorder or major depression (Rajkowska et al., 2001; Bowley et al., 2002) was also reported. Considering these reports, it is likely that indirect actions of THs through glial cells are important for neuronal activity and their impairment may at least in part, induce psychiatric symptoms.

These psychiatric symptoms can be seen in both hyperthyroidism and hypothyroidism. When it comes to the metabolism and balance of TH levels, it must be noted that propylthiouracyl (PTU) is a thiouracil-derived drug that inhibits thyroid peroxidase and type 1 deiodinase (D1), which is used to treat hyperthyroidism by decreasing TH level by suppression of T4 and T3 production. However, unlike D1 which is expressed mainly in the liver, kidney and testis, the major deiodinase D2 in the brain is known to be insensitive to PTU. Both mRNA concentration and activity of D2 are increased in hypothyroidism (van Doorn et al., 1982, 1983) and decreased in hyperthyroidism (Leonard et al., 1981; van Doorn et al., 1984; Croteau et al., 1996; Burmeister et al., 1997). D2 was also reported to be up-regulated in reactive astrocytes following traumatic brain injury (Zou et al., 1998). Thus, D2 is believed to serve a protective role to preserve the concentration of intracerebral T3 during states of thyroid hormone deficiency. This may explain, in part, why both hypo- and hyperthyroidism cause similar neurological symptoms.

## Conclusion

T3 is important not only for neuronal development but also for differentiation of astrocytes and oligodendrocytes, and for microglial development. In addition, T3 is an important signaling factor that affects microglial functions via complex mechanisms. Therefore, dysfunction of THs may impair glial function and thus disturb of the brain, which may cause mental disorders.

## Conflict of Interest Statement

The author declares that the research was conducted in the absence of any commercial or financial relationships that could be construed as a potential conflict of interest.
